# Two cases of gallbladder metastasis from renal cell carcinoma and review of literature

**DOI:** 10.1186/s12957-016-0843-3

**Published:** 2016-03-22

**Authors:** Mafalda Costa Neves, Kyriakos Neofytou, Alexandros Giakoustidis, Stephen Hazell, Andrew Wotherspoon, Martin Gore, Satvinder Mudan

**Affiliations:** Academic Surgery Department, The Royal Marsden NHS Foundation Trust, Fulham Road, London, SW3 6JJ UK; Surgery Department, The London Clinic, 116 Harley Street, London, W1G 7JL UK; Pathology Department, The Royal Marsden NHS Foundation Trust, Fulham Road, London, SW3 6JJ UK; Urology Department, The Royal Marsden NHS Foundation Trust, Fulham Road, London, SW3 6JJ UK; Department of Surgery and Cancer—Faculty of Medicine, Imperial College London, London, W2 1PG UK

**Keywords:** Cancer, Gallbladder, Kidney neoplasms, Clear cell metastatic renal cell carcinoma

## Abstract

**Background:**

Renal cell carcinoma accounts for 90 % of renal neoplasms and metastatic disease is common. One third of newly diagnosed cases will have synchronous metastases at diagnosis and further 25–50 % will develop metachronous disease.

**Case Presentation:**

This study presents two new cases of gallbladder metastasis from renal cell carcinoma (RCC) from our institution and reviews the published literature. The final cohort included 52 evaluable patients. M/F ratio was 2:1 and median age was 62.5 years. Most patients were diagnosed incidentally after follow-up or staging imaging for RCC. Of the patients with available histology, all except one were clear cell type (*n* = 39) and 92 % were polypoid. Thirty-six patients demonstrated metachronous gallbladder metastasis with median disease-free interval (DFI) from nephrectomy of 4 years. The most frequent site of metastasis was the contralateral kidney (46.7 %) followed by the pancreas and lung. The median disease-free interval (DFS) after cholecystectomy was 37 months. Three- and five-year OS rates were 74 and 62 %, respectively. Age younger than 45 years (*p* = 0.008) and DFI <24 months (*p* = 0.049) were associated with decreased OS.

**Conclusions:**

RCC metastasis to the gallbladder is associated with an unusual pattern of concomitant metastasis. Symptoms are not common. Simple cholecystectomy is associated with increased OS and nil local or port site recurrence. Young age and short DFI are associated with decreased OS.

**Electronic supplementary material:**

The online version of this article (doi:10.1186/s12957-016-0843-3) contains supplementary material, which is available to authorized users.

## Background

Renal cell carcinoma accounts for approximately 3 % of malignancies in adults and 90 % of renal neoplasms [1]. Metastatic disease is common, and one third of newly diagnosed cases will have synchronous metastatic disease at presentation. A further 25–50 % will develop metachronous disease [2]. Latent distant metastasis is characteristic of renal cell carcinoma (RCC) and can manifest more than a decade after nephrectomy in about 10 % of patients [3].

Clear Cell (CC) RCC is the most common type of renal cancer, accounting for 75 % of all primary kidney tumours. Similar to other malignancies, RCC may undergo differentiation to more aggressive histological types. Sarcomatoid transformation is present in 5 % of RCC, although these do not in themselves represent a distinct histological entity [4]. Spindle cell morphology, similar to sarcomas, can occur in all types of RCC but is most often seen in clear cell and chromophobe RCC [5]. These patients have an adverse stage stratified prognosis with a median survival of 4–19 months [6].

The most common sites of metastasis of RCC are the lung, bone, liver, adrenal gland, brain and contralateral kidney but have been documented in nearly every organ. Gallbladder involvement is reported in <1 % of cases. The outcome for metastatic RCC is poor, bearing a 5-year survival rate of approximately 5–10 %, but curative resection of metastasis in selected patients is known to improve long-term survival [4, 7].

### Materials and methods

The review of literature was based on a systematic PubMed search to identify all published cases of RCC metastasis to the gallbladder and including cases described in the Japanese literature. The final analysis included 52 patients, 2 from our institution and 50 from previous published cases (Table 1, database available in Additional file 1: Table S1). Four articles were excluded because of unavailability of content or omission of basic information particularly sex, age and interval between nephrectomy and the diagnosis of metachronous gallbladder lesion. When available, the data was collected to focus on demographics, histology and staging of the primary tumour, disease-free interval (DFI) from nephrectomy, size and extent of invasion into the gallbladder, presence of gallstones, disease-free survival, adjuvant therapy, follow-up time, and outcome.

**Table 1:** Review of literature

Authors	No. of cases	Source
Jain	1	Saudi J Kidney Dis Transpl 2013 Jan;24(1):100–4
Zygulska	1	Pol PrzeglChir 2012 Jun;84(6):313–6
Robledo	1	OncolLett 2012 May;3(5):1136–1138
Zevallos Quiroz	1	Cir Esp 2014 Apr;92(4):295–6
Decoene	1	Case Rep Med 2011;2011:671645
Chung	4	UrolOncol 2012 Jul–Aug;30(4):476–81
Kawahara	1	Case Rep Oncol 2010 Jan 29;3(1):30–34
Fang*.*	4	Arch Pathol Lab Med 2010 Jul;134(7):1003–9
Shoji	1	OncolLett 2010 May;1(3):507–509
Patel	1	Cases J. 2009 Oct 29;2:172
Kücükakin	1	UgeskrLaeger 2009 Aug 24;171(35):2486–7
Sand	1	Eur J Med Res 2009;14:90–2
Moujahid	1	GastroenterolClinBiol 2008 Aug–Sep;32(8–9):788–9
Nojima	1	J HepatobiliaryPancreatSurg 2008;15(2):209–12
Hellenthal	1	IntUrolNephrol 2007;39(2):377–9
Pandey	1	Indian J Gastroenterol 2006 May–Jun;25(3):161–2
Ishizawa	1	Asian J Surg2006 Jul;29(3):145–8
Miyagi	1	RinshoHinyokika 2003;57:257
Limani	1	ActaChirBelg 2003 Apr;103(2):233–4
Park	1	Yonsei Med J 2003 Apr 30;44(2):355–8
Gekiya	1	Jpn J UrolSurg2002;15:67
Aoki	2	Surg Today 2002;32(1):89–92
Ueki	1	Shoukakigazou 2001;3:373
Kechrid	1	Saudi J Kidney Dis Transpl 2000 Oct–Dec;11(4):587–92
Brasseur	1	J Radiol 1999 Jul;80(7):739–40
Celebi	1	Int J Urol 1998 May;5(3):288–90
Sparwasser	1	UrolInt 1997;58(4):257–8
Uchiyama	1	Jpn J Gastroenterol 1997;94:68
Furukawa	1	AJR 1997;169:1466
Kakimoto	1	HinyoukiGeka 1996;9:875
Lombardo	1	J Ultrasound Med 1996 Oct;15(10):725–8
Fujii	1	RinshoHinyokika 1995;49:405
King	1	Urology 1995 Nov;46(5):722–5
Pagano	1	Urology 1995 May;45(5):867–9
Coşkun	1	ActaChirBelg 1995;95:56
Naggler	1	Dig Dis Sci 1994 Nov;39(11):2476–9
Fullarton	1	Urology 1991 Aug;38(2):184–6
Golbey	1	Clin Imaging 1991 Oct–Dec;15(4):293–5
Satoh	1	Dig Dis Sci 1991 Apr;36(4):520–3
Terashima	1	Jpn J GastroenterolSurg 1990;23:1952
Harder	1	UgeskrLaeger 1983 Oct 17;145(42):3261
Oikawa	1	GekaShinryo 1978;20:617
Botting	1	Mayo ClinProc 1963;38:225
Costa Neves	2	Current cases
Total	52	

The two case reports presented resulted from a 5-year retrospective search through The Royal Marsden Hospital’s histopathological archives from gallbladder specimens. Data was collected through review of patient history, operative notes, pathology reports, and medical records after consent.

### Statistical analysis

Statistical analysis was performed with the Statistical Package of the Social Sciences (SPSS), version 17.0. The primary endpoints of the study were DFI between resection of primary renal tumour and presentation of metachronous gallbladder metastasis. Overall survival (OS) was calculated from the date of operation for the gallbladder metastasis to the date of disease-related death and was censored at the time of last follow-up for patients that were alive or at the time of death when unrelated to the disease. The impact of clinicopathological characteristics on OS was analysed using both the Kaplan-Meier and univariate Cox regression methods. Survival outcomes between groups were compared with the log-rank test.

A chi-square test, Mann-Whitney non-parametric test and Fischer’s exact test were used when appropriate for calculating the association between clinicopathological characteristics and interval between resection of primary renal tumour and presentation of metachronous gallbladder metastasis, recurrence or disease-related death. A *p* value of less than 0.05 was considered statistically significant.

### Case 1

In February 2012, a 60-year-old female presented with a urinary tract infection (UTI), right loin pain and haematuria. In spite of effective treatment of the UTI, the loin pain persisted. Evaluation by biphasic enhanced CT confirmed the presence of a 4-cm hypo-attenuating mass occupying the lower pole of the right kidney without invasion of the major renal vein or cava and without lymph node enlargement. A number of nodules highly suggestive of metastasis were present in both lungs, the largest measuring 6.5 mm. CA 19.9, CA 15.5 and CEA were negative. The patient underwent an uneventful laparoscopic right radical nephrectomy. Histology of the specimen showed CC RCC Leibovich score 1, pT1aN0 and Fuhrman grade 3.

**Fig. 1:**
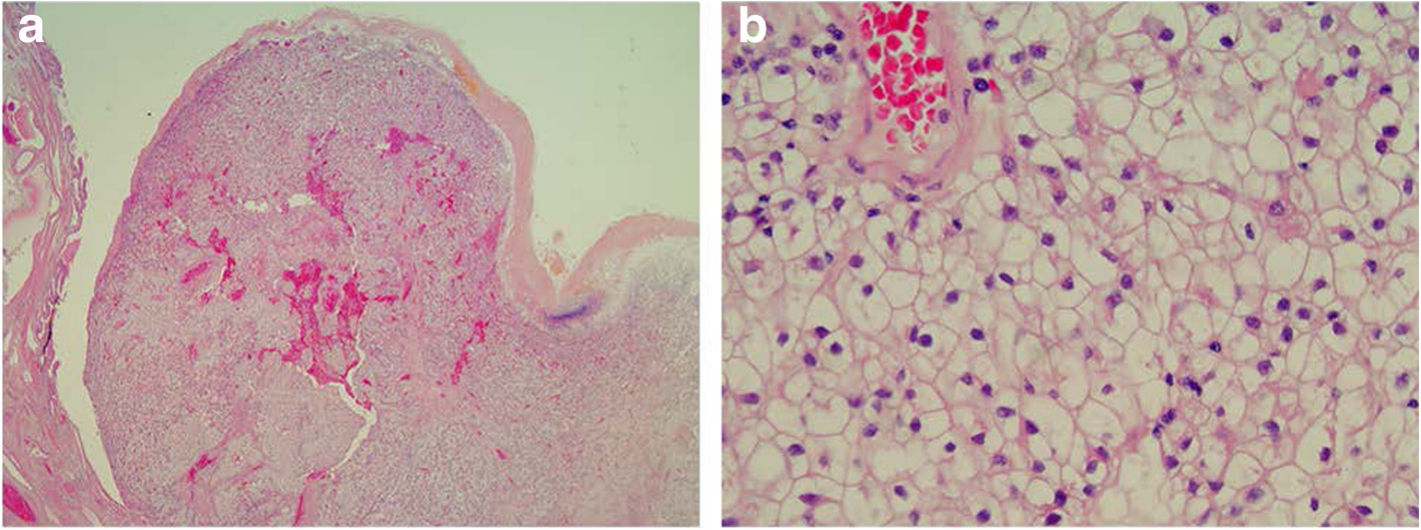
Gallbladder specimen in low (**a**) and high-power field (**b**) showing the polyp confined to the gallbladder and composed of large nests and sheets of moderately sized polygonal cells with delicate cell borders and clear cytoplasm

Follow-up CT scan after 2 months showed no recurrence and stability of the lung lesions, but the scan at month 5 showed total regression of the pulmonary nodules. Follow-up CTs every 6 months were unremarkable until July 2014, when a gallbladder mass was revealed. She had a further MRI, which showed a 2.5-cm intraluminal polypoid mass in the inferior wall of the gallbladder. A laparoscopic cholecystectomy was performed and the patient was discharged the following day. Histopathology reported a 30 × 20 mm polypoid submucosal metastatic deposit of CC RCC with an extensively ulcerated surface, composed of large nests and sheets of moderately sized polygonal cells with delicate cell borders and clear cytoplasm and central nucleus showing hyperchromasia without a prominent nucleolus (Fig. 1). The polyp was confined to the gallbladder with no extension into serosa. Immunohistochemistry showed the tumour cells to strongly express PAX8, Vimentin, CAM5.2, AMACR and EMA, focal E-cadherin expression with no tumour expression of CD10, RCCAg, CK7, or CD117.

The patient did not receive adjuvant therapy and remained disease-free after 2 months of follow-up.

### Case 2

A 57-year-old male with a previous history of resected melanoma from the right arm (T1a) presented with sudden onset of acute abdominal pain and macroscopic haematuria in May 2011. A CT scan showed a 12 × 10 × 8 cm mass in the left kidney extending into the hilum consistent with RCC and a gallbladder polyp. A number of bilateral small peripheral and sub-pleural pulmonary nodules measuring less than 5 mm and of indeterminate nature were present. In June 2011, the patient underwent an open radical nephrectomy and adrenalectomy, with an uneventful recovery. Pathology revealed a CC RCC Fuhrman 4 with extensive sarcomatoid changes and areas of necrosis, invading muscular veins of renal sinus including the main renal vein but without invasion of perinephric fat. All nodes were negative for malignancy, staging the tumour as pT3bN0.

The next month, the patient experienced two episodes of severe upper abdominal pain, nausea and vomiting which completely subsided after administration of intravenous morphine, accompanied by unremarkable CT emergency scans. He had a Barium swallow test and an upper gastrointestinal endoscopy, both of which were normal. On the third episode of acute abdominal pain in July, he finally underwent a laparoscopic cholecystectomy with relief of symptoms. Histopathology revealed a 22 × 15 × 8 mm polyp with normal mucosa composed of nested/alveolar pattern of moderate-sized polygonal cells with clear cytoplasm and well-defined cytoplasm borders, round central nuclei with a moderate degree of nucleolar prominence, occasionally multinucleated, with fine fibro-vascular septae present within thin wall vessels and an admixture of inflammation and foamy macrophages at the periphery of the polyp. Immunohistochemistry showed these cells to express AE1/AE3, Vimentin, CD10 and RCCAg with no expression of S100 or CEA, consistent with the diagnosis of metastatic CC RCC.

The patient was followed up in an outpatient clinic accompanied by CTs at 3, 6 and 12 months post-op and annually thereafter. The bilateral lung lesions remained stable without adjuvant treatment, and the patient remained disease-free until date (total follow-up of 38 months).

### Results of literature review

Patient’s relevant primary tumour and gallbladder metastasis characteristics are shown in Table 2. There was a predominance of male patients (67.3 %), and the median age at presentation of gallbladder metastasis was 62.5 years old (range 39–84). Of the 34 cases with initial staging available, 19 demonstrated stage 4 disease (55.9 %): 9 due to a single synchronous gallbladder metastasis, 7 with other synchronous metastasis and 3 with metachronous gallbladder involvement.

**Table 2:** Patient, primary tumour and gallbladder metastasis characteristics

Parameter	No. of patients (%) or median (range)	No. of patients available for analysis
Median age (years)	62.5 (39–84)	52/52
Sex		52/52
Male	35 (67.3)	
Female	17 (32.7)	
Stage of RCC at presentation		34/52
1	5 (14.7)	
2	4 (11.8)	
3	6 (17.6)	
4	19 (55.9)	
Timing		52/52
Synchronous	*16 (30.8)*	
Gallbladder only	9 (17.3)	
Gallbladder with other metastasis	7 (13.5)	
Metachronous	*36 (69.2)*	
Gallbladder only	21 (40.4)	
Gallbladder with other metastasis	14 (26.9)	
Unknown	1 (1.9)	
DFI, years	4 (0.25–27)	35/35
Presentation		39/52
Symptomatic	10 (25.6)	
Radiographic	29 (74.4)	
Median size of gallbladder tumour, cm	25 (8–75)	44/52
Extent of invasion of gallbladder		20/52
Mucosa	13 (65)	
Muscularis mucosa	4 (20)	
Subserosa	3 (15)	
Marcoscopic appearance		50/52
Polypoid/pedunculated	46 (92)	
Mass/nodule	4 (8)	
Presence of gallstones		45/52
No	38 (84.4)	
Yes	7 (15.6)	
Pathology		39/52
Clear cell RCC	38 (97.4)	
RCC type not specified	1 (2.6)	

Patients with synchronous and metachronous gallbladder metastases are highlighted in italic

In most of the cases, the diagnosis was made incidentally during a radiographic exam for staging or on follow-up (74.4 %, *n* = 39). When symptomatic, the majority of cases presented with acute or chronic biliary symptoms. Of the cases with available pathology of the gallbladder RCC metastasis, all except one were confirmed to be CC RCC, the remaining being classified as RCC “type not specified”. The gallbladder lesion was persistently polypoid/pedunculated and intraluminal in 92 % of cases. The mean size was 25 mm (range 8–75). Mucosal invasion was evident in 65 %, and gallstones were present in only 15.6 % (7/45).

The majority of patients (36/52–69.2 %) demonstrated metachronous gallbladder metastasis with a median DFI of 4 years (range 0.25–27). DFI was not predicted by sex, staging, incidental diagnosis vs. symptomatic disease, single vs. multiple metastases, macroscopic appearance of gallbladder tumour, its extent of invasion in the gallbladder wall or presence of gallstones.

The gallbladder was the sole site of metastatic disease in 30/52 patients, and in 70 % of these patients presented as a metachronous lesion. Patients with multiple sites of metastasis had the following pattern of disease: contralateral kidney (46.7 %), pancreas (26.7 %), lung (26.7 %), adrenal (20 %), skin (4.8 %), bones (4.8 %), IVC (4.8 %), ovaries (4.8 %), bladder (4.8 %) and muscle (4.8 %) (Table 3).

**Table 3:** Presentation of all sites of metastasis

Parameters	No. of patients (%) or median (range)	No. of patients available for analysis
Metastatic disease presentation		51/52
Metastasis to only gallbladder	30 (58.5)	
Metastasis to other sites	21 (41.2)	
Sites of other metastases		15/21
Contralateral kidney (%)	7 (46.7)^a^	
Pancreas (%)	4 (26.7)^a^	
Lung (%)	4 (26.7)^a^	
Adrenal (%)	3 (20)^a^	
Skin (%)	1 (4.8)^a^	
Bones (%)	1 (4.8)^a^	
IVC (%)	1 (4.8)^a^	
Ovaries (%)	1 (4.8)^a^	
Bladder (%)	1 (4.8)^a^	
Muscle (%)	1 (4.8)^a^	

^a^Percent of 15 patients with known sites of other metastasis

All patients had cholecystectomy and seven received adjuvant therapy.

**Fig. 2:**
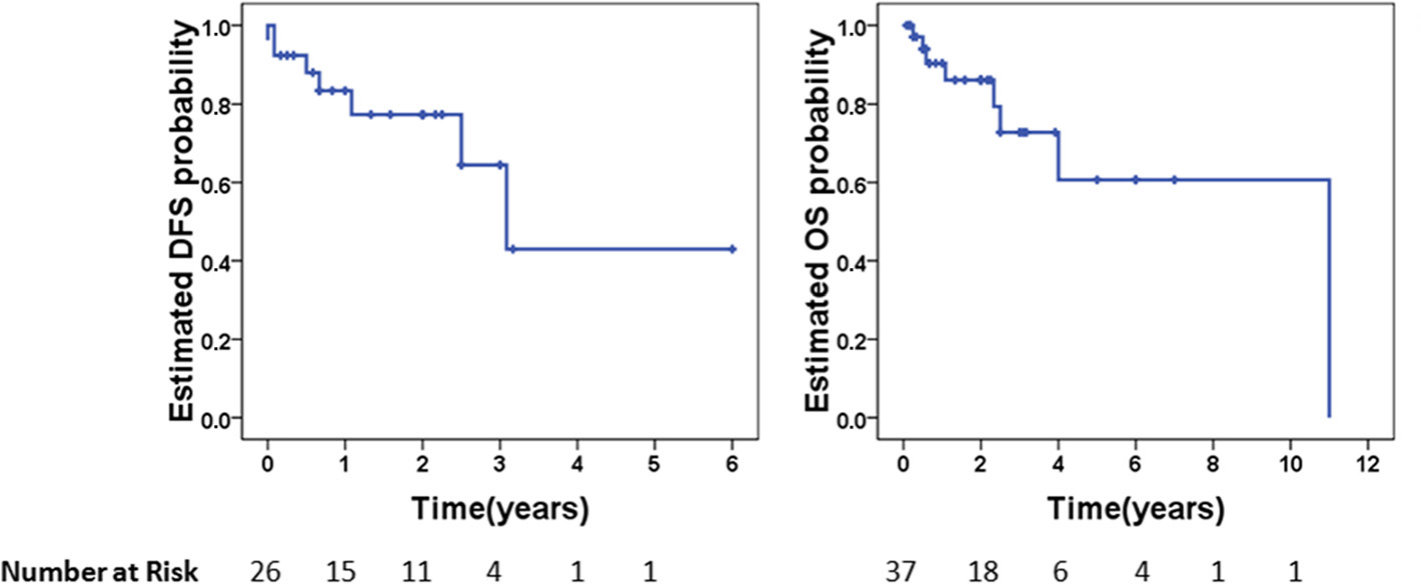
Kaplan-Meier curve of disease-free survival (DFS) and overall survival (OS)

At a mean follow-up of 1.6 years (range 0.1–11) after cholecystectomy, 52.6 % of the patients were alive with no evidence of disease, 21.1 % were alive with disease, 21.1 % died from metastatic disease and 5.3 % died from unrelated causes (Table 4). The median disease-free interval (DFS) was 37 months (95 % confidence interval 24–48). Three- and five-year OS rates were 74 and 62 %, respectively (Fig. 2). No patients developed local or port site recurrence after simple cholecystectomy.

**Table 4:** Intervention, follow-up and outcome

Parameters	No. of patients (%)	No. of patients available for analysis
Intervention		
Cholecystectomy	51 (100)	51/52
Adjuvant therapy	7 (25.9)	27/52
Median follow-up time, years, median (range)	1.6 (0.1–11)	37/52
5-year OS, %	62	37/52
Median DFS, months (95 % CI)	37 (24–48)	26/52
Available outcome		38/52
No evidence of disease	20 (52.6)	
Alive with metastasis	8 (21.1)	
Death from metastasis	8 (21.1)	
Non-cancerous death	2 (5.3)	

Abbreviations: *OS* overall survival, *DFS* disease-free survival, *CI* confidence interval

**Fig. 3:**
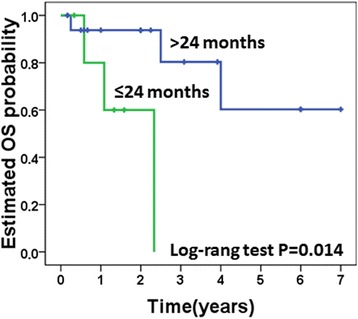
Disease-free survival (DFI) between nephrectomy and diagnosis of gallbladder metastasis and overall survival (OS) in patients with metachronous gallbladder metastasis

**Fig. 4:**
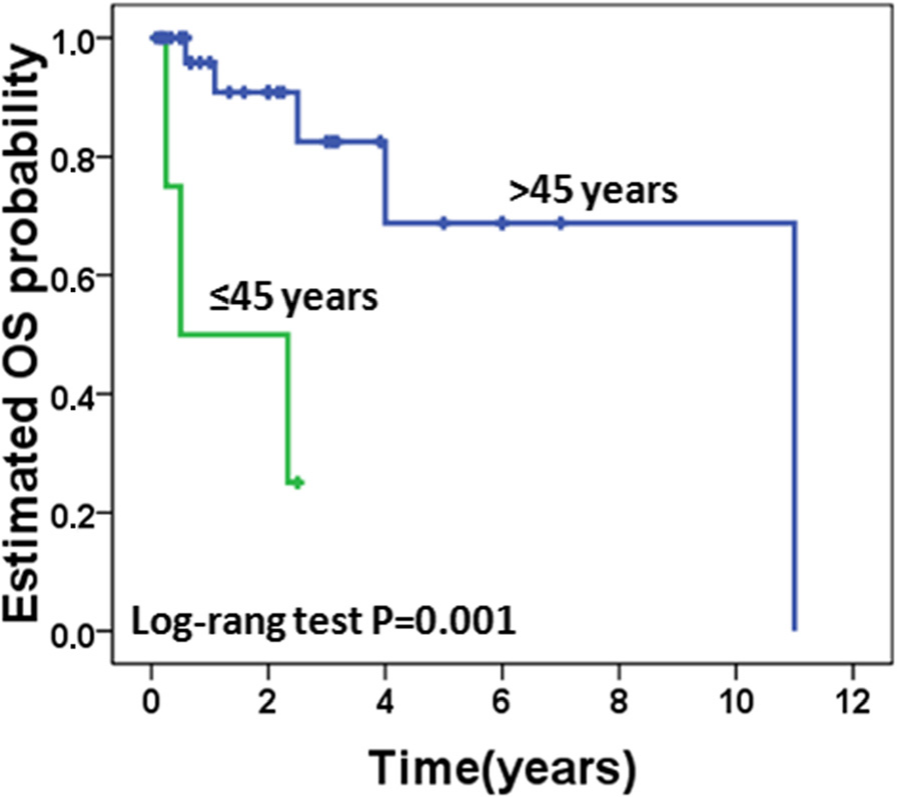
Age at diagnosis of gallbladder metastasis and overall survival (OS)

Univariate analysis (Table 5) revealed that age less than 45 years (hazards ratio (HR) 8.98; 95 % CI, 1.78–45.18; *p* = 0.008) and DFI <24 months (HR 10.25; 95 % CI, 1.01–103.67; *p* = 0.049) were associated with decreased OS (Figs. 3 and 4).

**Table 5:** Baseline clinicopathologic characteristics and their association with OS in univariate analysis

Parameters	No. of patients (%)	HR (95 % CI)	*p* value	No. of patients available for analysis
Sex				37/52
Male	25 (67.6)	1 (referent)		
Female	12 (32.4)	1.37 (0.30–6.25)	0.682	
Age				37/52
>45	33 (89.2)	1 (referent)		
<45	4 (10.8)	8.98 (1.78–45.18)	*0.008*	
Presentation				28/42
Asymptomatic	7 (25)	1 (referent)		
Symptomatic	21 (75)	2.97 (0.59–14.77)	0.183	
Timing				37/52
Synchronous^a^	14 (37.8)	1 (referent)		
Metachronous	23 (62.2)	3.25 (0.39–27.10)	0.276	
Staging				28/52
1	2 (7.1)	^b^		
2	4 (14.3)	1 (referent)		
3	5 (17.9)	0.56 (0.05–6.33)		
4	17 (60.7)	0.74 (0.30–1.83)	0.676	
Single vs. multiple				36/52
Single	20 (55.6)	1 (referent)		
Multiple	16 (44.4)	2.77 (0.50–15.28)	0.241	
Interval from resection of primary to diagnosis of metastasis				23/35^c^
>24 months	17 (73.9)	1 (referent)		
<24 months	6 (26.1)	10.80 (1.07–108.34)	*0.043*	
Adjuvant chemotherapy				
Yes	6 (28.6)	1 (referent)		21/52
No	15 (71.4)	2.28 (0.25–20.58)	0.461	

Abbreviations: *HR* hazards ratio, *CI* confidence interval

^a^Diagnosis at the time of diagnosis of primary tumour

^b^All cases are censored

^c^Only

Statistically significant *p*value is highlighted in italic

## Discussion

Despite the great tendency of RCC to metastasize synchronously or metachronously to numerous anatomical sites, metastasis to the gallbladder is rare. Our review of literature found 54 cases published by 43 papers dated since 1963. Fifty cases were considered evaluable for this report and with the addition of our two cases provided a total cohort of 52 evaluable cases, thus representing the most extensive and complete database on this subject to date.

Unlike primary gallbladder cancer, RCC metastases to the gallbladder show a clear male predominance and a low incidence of concomitant gallstone disease [8, 9]. Moreover, the majority has a polypoid or pedunculated morphology (46/50) whereas primary gallbladder cancer has a diffuse wall thickening morphology [9].

The median DFI was 4 years, which is consistent with the results found by Chung et al. in their review in 2012 [10]. We found a statistically significant decrease in OS for patients younger than 45 years old and when the DFI was less than 24 months.

The clear cell type was responsible for practically all cases of metastasis of RCC to the gallbladder. It is still not clear if this is due to the fact that CC RCC is the most frequent type of RCC or whether this type of metastasis is exclusive to clear cell type. A sarcomatoid component generally implies a more aggressive behaviour and bears a median survival of 4–19 months after diagnosis [6], but our second case patient had no recurrence throughout a follow-up period of 38 months despite sarcomatoid histology.

The diagnosis of gallbladder lesion was predominantly radiographic at initial staging or at follow-up after nephrectomy. The management of gallbladder polyps remains controversial, but a review of literature published in 2012 stated that gallbladder polyps >10 mm, symptomatic, fast growing, sessile or wide-based; polyps with long pedicles; any size of polyp in patients >50 years of age; concurrent gallstones; polyps in the setting of primary sclerosing cholangitis; or abnormal gallbladder ultrasound are considered indications for surgery [11]. In the context of patients with a history of RCC, the suspicion of a metastasis should be considered.

A simple cholecystectomy is recommended for pedunculated tumours because gallbladder cancers of pedunculated type often remain within the mucosa, regardless of the tumour size [12]. In our review, no patients with RCC who underwent a simple cholecystectomy for a metastatic polypoid lesion developed local recurrence suggesting that a simple cholecystectomy is adequate when a metastasis of RCC is suspected. Laparoscopic cholecystectomy followed by a frozen section is appropriate when there is no evidence of serosal involvement at laparoscopy [11], as port site metastases from gallbladder cancer have been reported [13].

It is interesting to notice that the presence of concomitant metastasis is different when there is gallbladder involvement. In our cohort, involvement of the contralateral kidney was the most frequent site of concomitant disease, exhibiting a frequency of 46.7 %, contrasting with the reported 5 % when gallbladder involvement is absent [14]. Pancreatic and lung involvement showed a frequency of 26.7 % each. This is intriguing, as the lung is usually the most frequent site of RCC metastasis, occurring in up to 60 % of patients [15], and pancreatic involvement is reported in only 2 % of cases [16].

In our first case report, small bilateral lung lesions were identified and the CT suggested these to be metastatic disease. However, these lesions regressed completely 5 months after nephrectomy without adjuvant treatment. The nature of these lesions remains uncertain, because there was no tissue pathology confirmation of metastatic disease. The initial stability of the lesions and posterior total regression point towards a more probable inflammatory nature, in spite of the absence of respiratory symptoms, but spontaneous regression of lung metastasis from RCC after nephrectomy is reported [17, 18]. In our second case report, the patient also demonstrated bilateral small lung lesions, some of them described as inflammatory on CT. These lesions never regressed but also did not progress throughout a 38-month follow-up, suggesting the diagnosis of lung metastasis as unlikely.

Several phase 3 randomized controlled studies are currently in place for adjuvant treatment with sunitinib, sorafenib, pazopanib, axitinib and everolimus. As the results from these trials have not been published yet, there is still no current evidence to support adjuvant treatment after nephrectomy for RCC [1, 4].

## Conclusions

Gallbladder metastasis from RCC is an uncommon event. Most cases are asymptomatic, hence the importance of radiological follow-up after nephrectomy. All gallbladder polypoid lesions in patients with a history of RCC mandate treatment, and simple cholecystectomy is associated with increased OS and nil local or port site recurrence published to date. The presence of gallbladder metastasis indicates an alteration in the pattern of subsequent disease with relapse in the contralateral kidney being the most common site of recurrence. Young age and short DFI are associated with decreased OS.

### Consent

Written informed consent was obtained from the patients for publication of these case reports and any accompanying images. Copies of the written consent are available for review by the Editor-in-Chief of this journal.

## Additional file

Additional file 1:
**Table S1.** Literature review. (DOCX 34 kb)
