# Supplementary material for: Two cases of gallbladder metastasis from renal cell carcinoma and review of literature
Source: World J Surg Oncol. 2016 Mar 22;14:87. doi: 10.1186/s12957-016-0843-3 (PMC4802731; doi:10.1186/s12957-016-0843-3)
Supplement: Additional file 1: — Table S1. Literature review. (DOCX 34 kb) [file 12957_2016_843_MOESM1_ESM.docx]

| **Table 1. Literature review** | | | | | | | | | | | | | | | | |
| --- | --- | --- | --- | --- | --- | --- | --- | --- | --- | --- | --- | --- | --- | --- | --- | --- |
| **Author** | **Age/ Sex** | **Presentation** | **Staging** | **Single / Multiple** | **Synchronous/**  **Metachronous** | **DFI (months)** | **Macroscopic appearance** | **Type** | **Size** | **Stones** | **Disease free (months)** | **Follow up (months)** | **Future metastasis** | **Adjuvant QT** | **Outcome** | **Source** |
| **Jain** | 49/F | Chronic Cholecystitis | Stage I | S | Meta | 72 | Polypoid | CC | 14,5 | Y |  | NA |  | N | AWD | [*Saudi J Kidney Dis Transpl*](http://www.ncbi.nlm.nih.gov/pubmed/?term=Jain+D+and+Chopra+B%2C+Metastatic+renal+cell+carcinoma+of+gall+bladder.) 2013 Jan;24(1):100-4 |
| **Zygulska** | 70/F | Radiographic | Stage II | S | Meta | 32 | Polypoid | CC | 8 | Y | 13 | 47 | Y | N | AD | [*Pol PrzeglChir*](http://www.ncbi.nlm.nih.gov/pubmed/?term=Zygulska+AL%2C+W%C3%B3jcik+A%2C+Richter+P%2C+Krzesiwo+K.Renal+carcinoma+metachronous+metastases+to+the+gall-bladder+and+pancreas) 2012 Jun;84(6):313-6 |
| **Robledo** | 75/F | Radiographic | Stage III | M | Meta | 6 | Polypoid | CC | 20 | N | 4 | 4 | N | N | AWD | [*OncolLett*](http://www.ncbi.nlm.nih.gov/pubmed/?term=Robledo+AM%2C+Millet+SB%2C+Orbis+Castellanos+JF%2C+et+al.+Metastasis+of+the+gallbladder+in+clear+cell+renal+carcinoma.) 2012 May;3(5):1136-1138 |
| **Zevallos Quiroz** | 55/F | Radiographic |  | M | Meta | 72 | Solid nodule | CC | 35 | N |  |  |  | N |  | [*Cir Esp*](http://www.ncbi.nlm.nih.gov/pubmed/?term=Zevallos+Quiroz+JC%2C+Lizarazu+P%C3%A9rez+A%2C+Guisasola+Gorrochategui%2C+et+al.+Gallbladder+metastasis+from+renal+cell+carcinoma%2C+an+extremely+rare+neoplastic+dissemination+location) 2014 Apr;92(4):295-6 |
| **Decoene** | 63/F | Radiographic | Stage I | M | Meta | 192 | Polypoid | CC | 19 | N |  |  |  | N |  | [*Case Rep Med*](http://www.ncbi.nlm.nih.gov/pubmed/?term=Decoene+J%2C+Ameye+F%2C+Lerut+E%2C+et+al.Renal+cell+carcinoma+with+synchronous+metastasis+to+the+calcaneus+and+metachronous+metastases) 2011;2011:671645 |
| **Chung** | 63/F | Radiographic | Stage III | S | Meta | 120 | Polypoid | CC | 35 |  |  | 84 | Y | N | AD | [*UrolOncol*](http://www.ncbi.nlm.nih.gov/pubmed/?term=Renal+Cell+Carcinoma+with+Metastases+to+the+Gallbladder%3A+Four+Cases+from+the+National+Cancer+Institute+(NCI)+and+Review+of+the+Literature) 2012 Jul-Aug;30(4):476-81 |
|  | 52/F | Radiographic | Stage IV | S | Syn | 0 | Polypoid | CC | 18 |  |  | 60 | Y | N | AD |  |
|  | 51/M | Radiographic | Stage IV | M | Syn | 0 | Polypoid | CC | 17 |  |  | 132 | Y | Y | DFD |  |
|  | 42/M | Radiographic | Stage IV | S | Syn | 0 | Polypoid | CC |  |  |  | 6 | Y | N | DFD |  |
| **Kawahara** | 73/F | Radiographic | Stage IV | M | Syn | 0 | Polypoid | CC | 10 | N |  |  |  | N |  | [*Case Rep Oncol*](http://www.ncbi.nlm.nih.gov/pubmed/?term=Kawahara+T%2C+Ohshiro+H%2C+Sekigushi+Z%2C+et+al.+Gallbladder+Metastasis+from+Renal+Cell+Carcinoma.) 2010 Jan 29;3(1):30-34 |
| **Fang** | 45/M | Radiographic | Stage IV | M | Meta | 14 | Polypoid | CC | 19 | Y | 8 | 28 | Y | Y | DFD | [*Arch Pathol Lab Med*](http://www.ncbi.nlm.nih.gov/pubmed/?term=Intraluminal+polypoid+metastasis+of+renal+cell+carcinoma+in+gallbladder+mimicking+gallbladder+polyp) 2010 Jul;134(7):1003-9 |
|  | 65/F | RUQ pain | Stage IV | M | Meta | 15 | Polypoid | CC | 25 | N |  | 7 | Y | N | DFD |  |
|  | 54/M | Radiographic | Stage III | S | Meta | 84 | Polypoid | CC | 15 | N | 27 | 27 | N | N | AWD |  |
|  | 51/M | Radiographic | Stage III | M | Meta | 84 | Polypoid | CC | 17 | N | 37 | 37 | Y | Y | AD |  |
| **Shoji** | 50/M | Radiographic | Stage I | M | Meta | 46 | Polypoid | CC | 11 | N | 8 | 8 | N | N | AWD | [*OncolLett*](http://www.ncbi.nlm.nih.gov/pubmed/?term=Shoji+S%2C+Mukai+M%2C+Yazawa+N%2C+et+al.+Metastasis+to+gallbladder+and+adrenal+gland+of+renal+cell+carcinoma.) 2010 May;1(3):507-509 |
| **Patel** | 64/F | Chronic Cholecystitis |  | S | Meta | 72 | Polypoid | CC | 30 | Y |  |  |  | N |  | [*Cases J.*](http://www.ncbi.nlm.nih.gov/pubmed/?term=Patel+S%2C+Zebian+B%2C+Gurjar+S%2C+et+al.+An+unusual+gall-bladder+polyp--site+of+metastatic+renal+cell+carcinoma%3A+a+case+report.) 2009 Oct 29;2:172 |
| **Kücükaki** | 80/M | Radiographic |  | S | Meta | 12 |  | CC |  |  |  |  |  |  |  | *UgeskrLaeger* 2009 Aug 24;171(35):2486-7 |
| **Sand** | 48/F | RUQ pain | Stage IV | M | Meta | 60 | Polypoid | CC | 25 | N | 0 | 72 | Y | Y | AD | [*Eur J Med Res*](http://www.ncbi.nlm.nih.gov/pubmed/19258219) 2009;14:90-2 |
| **Moujahid** | 56/M | Chronic Cholecystitis | Stage IV | S | Syn | 0 | Polypoid | CC |  | N | 8 | 8 | N | N | AWD | [*GastroenterolClinBiol*](http://www.ncbi.nlm.nih.gov/pubmed/?term=Moujahid+M%2C+Zrara+I%2C+Touiti+D%2C+Daali+M.+Renal+cell+carcinoma+with+gallbladder+metastasis.) 2008 Aug-Sep;32(8-9):788-9 |
| **Nojima** | 61/M | Radiographic | Stage IV | S | Syn | 0 | Polypoid | CC | 15 | N | 10 | 10 | N | N | AWD | [*J HepatobiliaryPancreatSurg*](http://www.ncbi.nlm.nih.gov/pubmed/?term=Renal+cell+carcinoma+with+unusual+metastasis+to+the+gallbladder+nojima) 2008;15(2):209-12 |
| **Hellenthal** | 39/M | Radiographic | Stage IV | S | Syn | 0 | Polypoid | CC | 40 | N | 30 | 30 | N | N | AWD | [*IntUrolNephrol*](http://www.ncbi.nlm.nih.gov/pubmed/?term=Renal+cell+carcinoma+metastatic+to+gallbladder%3A+a+survival+advantage+to+simultaneous+nephrectomy+and+cholecystectomy) 2007;39(2):377-9 |
| **Pandey** | 46/M | Non-specific abdominal pain |  | S | Meta | 11 | Polypoid | CC |  |  | 16 | 16 | N |  | AWD | [*Indian J Gastroenterol*](http://www.ncbi.nlm.nih.gov/pubmed/?term=pandey+gallbladder+renal+cell) 2006 May-Jun;25(3):161-2 |
| **Ishizawa** | 73/M | Radiographic | Stage II | S | Meta | 60 | Pedunculated | CC | 35 | N | 24 | 24 | N | Y | AWD | [*Asian J Surg*](http://www.ncbi.nlm.nih.gov/pubmed/16877212)2006 Jul;29(3):145-8 |
| **Miyagi** | 53/M |  |  | S | Meta | 126 | Polypoid |  | 25 | N | 24 | 24 | N |  | AWD | *RinshoHinyokika* 2003;57:257 |
| **Limani** | 64/M | Radiographic |  | S | Meta | 12 | Polypoid |  | 15 | N |  |  |  |  |  | [*ActaChirBelg*](http://www.ncbi.nlm.nih.gov/pubmed/?term=limani+gallbladder+renal+cell) 2003 Apr;103(2):233-4 |
| **Park** | 48/M | Radiographic | Stage III | S | Meta | 24 | Polypoid | CC |  | N |  |  | Y | Y |  | [*Yonsei Med J*](http://www.ncbi.nlm.nih.gov/pubmed/12728482) 2003 Apr 30;44(2):355-8 |
| **Gekiya** | 68/M |  |  | S | Meta | 180 | Polypoid |  |  | N | 12 | 12 | N |  | AWD | *Jpn J UrolSurg*2002;15:67 |

| **Aoki** | 63M | Radiographic |  | S | Meta | 324 | Pedunculated | CC | 75 | N | 72 | 72 | N |  | AWD | [*Surg Today*](http://www.ncbi.nlm.nih.gov/pubmed/?term=aoki+gallbladder+renal+cell)*2002;32(1):89-92* |
| --- | --- | --- | --- | --- | --- | --- | --- | --- | --- | --- | --- | --- | --- | --- | --- | --- |
|  | 80M | Radiographic |  | S | Meta | 84 | Pedunculated | CC | 45 | N | 24 | 24 | N |  | AWD |  |
| **Ueki** | 69/F |  | Stage IV | S | Syn | 0 | Pedunculated |  | 16 | N | 7 | 7 | N |  | AWD | *Shoukakigazou 2001;3:373* |
| **Kechrid** | 55/F | RUQ pain | Stage II | M | Meta | 96 |  | CC |  | N | 30 | 30 | Y | N | DFD | [*Saudi J Kidney Dis Transpl*](http://www.ncbi.nlm.nih.gov/pubmed/?term=kechrid+gallbladder+renal+cell)*2000 Oct-Dec;11(4):587-92* |
| **Brasseur** | 69/M | Radiographic | Stage III | S | Meta | 7 | Polypoid | CC | 19 | N | 6 | 13 | Y | N | DFD | [*J Radiol*](http://www.ncbi.nlm.nih.gov/pubmed/?term=Brasseur+P%2C+Bissen+L%2C+Dupont+H%2C+Sukkarieh+F.+Metastasis+to+the+gallbladder+secondary+to+a+clear+cell+renal+tumor.)*1999 Jul;80(7):739-40* |
| **Celebi** | 73/M | Indicental | Stage IV | S | Syn | 0 | Polypoid | CC | 28 | N | 1 | 1 | Y |  | DFO | [*Int J Urol*](http://www.ncbi.nlm.nih.gov/pubmed/?term=celebi+gallbladder+renal+cell)*1998 May;5(3):288-90* |
| **Sparwasser** | 46/M |  | Stage II |  | Meta | 44 | Polypoid | RCC TNS |  | N |  | 48 | Y |  | DFD | [*UrolInt*](http://www.ncbi.nlm.nih.gov/pubmed/?term=sparwasser+gallbladder+renal+cell)*1997;58(4):257-8* |
| **Uchiyama** | 64/M |  |  | M | Meta | 36 | Pedunculated |  | 19 | N |  | 7 |  |  | AD | *Jpn J Gastroenterol 1997;94:68* |
| **Furukawa** | 41/M | Incidental |  | M | Meta | 3 | Polypoid |  | 19 | N |  |  |  |  |  | *AJR 1997;169:1466* |
| **Kakimoto** | 53/M |  |  | M | Meta | 48 | Polypoid |  | 15 | N |  | 6 |  |  | AD | *HinyoukiGeka 1996;9:875* |
| **Lombardo** | 77/M |  |  | S | Meta | 60 | Polypoid | CC | 30 | N |  |  | N |  |  | [*J Ultrasound Med*](http://www.ncbi.nlm.nih.gov/pubmed/?term=lombardo+gallbladder+renal+cell)*1996 Oct;15(10):725-8* |
| **Fujii** | 69/M |  | Stage IV | M | Syn | 0 | Polypoid |  | 28 | N | 3 | 3 | N |  | AWD | *RinshoHinyokika 1995;49:405* |
| **King** | 64/M | Incidental | Stage IV | S | Syn |  | Polypoid | CC | 35 | N | 26 | 26 | N |  | AWD | [*Urology*](http://www.ncbi.nlm.nih.gov/pubmed/7495130)*1995 Nov;46(5):722-5* |
| **Pagano** | 62/M | Incidental | Stage IV | M | Syn |  | Mass | CC | 35 | Y | 36 | 36 |  |  | AWD | [*Urology*](http://www.ncbi.nlm.nih.gov/pubmed/?term=pagano+gallbladder+renal+cell)*1995 May;45(5):867-9* |
| **Coşkun** | 52/M |  | Stage IV | M | Syn | 0 | Polypoid |  | 35 | N |  |  |  |  |  | *ActaChirBelg 1995;95:56* |
| **Naggler** | 82/M | Asymptomatic | Stage I | S | Meta | 60 | Polypoid | CC | 30 | Y |  |  | N |  |  | [*Dig Dis Sci*](http://www.ncbi.nlm.nih.gov/pubmed/?term=naggler+gallbladder+renal+cell)*1994 Nov;39(11):2476-9* |
| **Fullarton** | 43/F | Haemobilia and anemia |  | M | Meta | 324 | Mass | CC | 30 | N |  | 3 | Y |  | DFD | [*Urology*](http://www.ncbi.nlm.nih.gov/pubmed/?term=Fullarton+and+Burgoyn+gallbladder+renal+cell)*1991 Aug;38(2):184-6* |
| **Golbey** | 84/M | Acute cholecystitis |  | S | Meta | 156 | Two polyps | CC | 35 | N |  |  | N |  |  | [*Clin Imaging*](http://www.ncbi.nlm.nih.gov/pubmed/?term=golbey+gallbladder+renal+cell)*1991 Oct-Dec;15(4):293-5* |
| **Satoh** | 71/M | Radiographic |  | M | Meta | 12 | Polypoid | CC | 40 | N | 19 | 19 | N |  | AWD | [*Dig Dis Sci*](http://www.ncbi.nlm.nih.gov/pubmed/?term=satoh+gallbladder+renal+cell)*1991 Apr;36(4):520-3* |
| **Terashima** | 61/M |  | Stage IV | M | Syn | 0 | Mass |  | 20 | Y |  | 2 |  |  | DFO | *Jpn J GastroenterolSurg*  *1990;23:1952* |
| **Harder** | 66/M |  |  | S | Meta | 19 | Pedunculated |  | 42 |  |  |  |  |  |  | [*UgeskrLaeger*](http://www.ncbi.nlm.nih.gov/pubmed/?term=Harder+VV%2C+Heindorff+H.+Metastasis+from+renal+cell+carcinoma+to+the+gallbladder.)*1983 Oct 17;145(42):3261* |
| **Oikawa** | 70/F |  | Stage IV | M | Syn | 0 | Polypoid |  | 30 | N |  | 6 |  |  | AD | *GekaShinryo 1978;20:617* |
| **Botting** | 66/M |  |  | S | Meta | 19 | Pedunculated |  | 42 | N |  |  |  |  |  | *Mayo ClinProc 1963;38:225* |
| **Costa Neves** | 60/F | Radiographic | Stage I | S | Meta | 28 | Polypoid | CC | 30 | N | 2 | 2 | N | N | AWD | *Current cases* |
|  | 57/M | Acute biliary symptoms | Stage IV | S | Syn | 0 | Polypoid | CC | 22 | N | 38 | 38 | N | N | AWD |  |
| DFI – Disease free interval; QT – Chemotherapy; RUQ – Right upper quadrant; CC – Clear cell; TNS – Type not specified; AWD – Alive without disease; AD – Alive with disease; DFD – Dead from disease; DFO – Dead from other causes. | | | | | | | | | | | | | | | | |
